# Optimization of series-series compensated wireless power transfer system using alternative secondary side rectification

**DOI:** 10.1038/s41598-023-49305-9

**Published:** 2024-01-12

**Authors:** Martin Zavrel, Vladimir Kindl, Michal Frivaldsky, Darius Andriukaitis, Dangirutis Navikas

**Affiliations:** 1 Faculty of Electrical Engineering, West Bohemian University, Pilsen, Czech Republic; 2 Faculty of Electrical Engineering and Information Technologies, University of Zilina, Zilina, Slovakia; 3 Faculty of Electrical and Electronics, Kaunas University of Technology, Kaunas, Lithuania

**Keywords:** Electrical and electronic engineering, Power distribution

## Abstract

This paper focuses on the operational analysis of wireless power transfer (WPT) system, while the topology of the secondary side rectifier represents the main element, for which the properties of WPT system are being investigated. Initially the system description and technical specifications are given. Because WPT systems are designed for a certain type and value of the load (impedance matching) in order to achieve the highest possible efficiency, the definitions for those values are identified for individual topologies of the secondary side rectifiers. Consequently, the results are compared and discussed and followed by the simulation analysis to prove the operational behavior in time-domain for each of investigated alternative of rectifier. Several relationships have been identified in relation to secondary side electrical variables, and discussion for stress-optimization are given as well. The simulation results are verified by the experimental measurements, while individual solutions for secondary side rectifiers are evaluated from efficiency point of view followed by the recommendations of the operational conditions.

## Introduction

The global trend of mobile devices, rapid advances in electric vehicles, and the deployment of smart grids that require millions of energy-saving sensors have put wireless technology back in the spotlight. Unlike Information and Communication Technology (ICT), which requires small amounts of energy to transmit useful data, the goal of WPT is to safely transmit large amounts of electrical energy over a given distance to a load. In recent decades, the use of battery-powered devices such as mobile phones, electric vehicles, and medical implants has increased significantly around the world, and WPT can find significant applications.

WPT is used in a variety of industrial applications and robotics, such as transferring electrical energy through bent joints without physical contact^1–4^, and robotic devices performing tasks such as disaster relief in inaccessible or hazardous locations. It can also be used for applications^5–8^. For these reasons, there is a need to develop more efficient and secure designs for WPT technology. The purpose of this paper is to provide an up-to-date overview of current WPT topologies and highlight the limitations of the most commonly used technology, inductive power transfer systems, through simulations and hands-on analysis.

It is common knowledge that in order to transfer actual power to the load side of an IPT system, the leakage inductors of a loosely coupled transformer (LCT) must be corrected. To compensate the primary side and/or secondary side, a number of compensation networks have been proposed. Their major objectives are to attain or enhance the good traits listed below^9–13^: an output with a constant current and voltage that is unaffected by load resistance; zero voltage switching (ZVS), which can achieve great efficiency by soft switching, requires a weak inductive input impedance; Less sensitivity to the fluctuating coupling coefficient, which significantly broadens the range of applications.

In an IPT system, it is generally challenging for a compensation circuit to satisfy all the requirements. Tolerance for IPT namely, serial-serial (SS), serial-parallel (SP), parallel-serial (PS), and parallel-parallel (PP), are widely used in many scenarios. The SS compensation architecture, which achieves zero voltage switching and nearly zero reactive power without experiencing bifurcation occurrences, has more desirable properties than the other three topologies. For SS topology, only two compensating capacitors are required, which leads to less power loss, smaller size, higher power density, and lower cost. The performance of the converter's transfer, however, is almost fixed after the transformer has been identified unless a new LTC is substituted. Low design freedom and high sensitivity to misalignment highly restrict the practical promotion of SS-compensated IPT system.

Higher order compensation techniques, such LCL compensation topology, are suggested^14–16^ as a solution to the issue of ultra-large current in SS topology. Inverter and resonant tank power exchange is balanced by the additional compensating inductance. By using magnetic coupling, the primary coil current behaves as a current source and gives the secondary side a steady voltage. The LCC compensation topologies provide many desirable performances, such as (Zero Phase Angle) ZPA operations, high freedom of design, but more resonant elements, resulting in complex tune and the increase of system size and cost.

Once the practical and/or commercial use is under consideration the cost, size efficiency and power performance are the main issues, which are defining the qualitative indicators of the system. It is therefore desirable to utilize trusted, reliable, and robust solutions. This paper focuses on the standard SS compensated WPT system, while the analysis is related to the properties investigation of the secondary side rectifier configurations. The common bridge diode rectifier is compared to alternative solutions. First it is discussed about the impedance matching requirement, so for this purpose the determination of the reflecting AC side resistance is provided. Consequently, the operational properties of certain rectifier types are evaluated through simulation analysis in time domain followed by the performance investigation through laboratory measurements. Individual rectifier configurations are evaluated from efficiency point of view, while the recommendations for the target application use are given at the end of the paper.

## Materials and methods

Wireless power transfer systems are being under development for more than a decade. Through the time, many configurations of compensation network have been analyzed, while various application purposes have been identified as well. This research is focused on the series-series (SS) compensated WPT system, which is well suited to the application area of high-power vehicle battery chargers due to their electrical and transfer properties. SS compensated WPT system, similarly to other configurations, has certain specifications which must be considered when designing a WPT system for defined parameters of target application. The correct system settings, or impedance matching considering the type of the load is crucial according to achieve optimal operating parameters, especially efficiency and performance. Here, one of the important issues, which is affecting this performance is the type of the used secondary side rectifier^17–21^.

**Figure 1:**
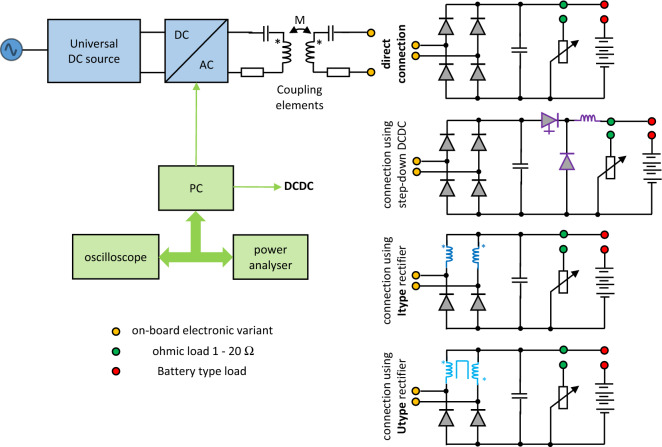
Block diagram and principal schematic of researched WPT system alternatives.

The investigated system can be illustrated using Fig. 1, which shows the input WPT system supply source, the WPT inverter, the coupling elements in series-series topology and power electronics of the on-board part of the secondary side system. Because presented research is focused on the optimization of the WPT system performance using alternative configuration of secondary side rectifier, four topologies as a direct connection to the load (resistor or battery) are considered.

- Standard bridge diode rectifier,
- Diode rectifier in combination with a DC-DC converter,
- Diode rectifier with current output (I_type_),
- Diode rectifier with voltage output (U_type_).

The entire system control and data acquisition from measurements are supervised by the PC equipped by relevant control algorithm. The parameters of individual core components are listed in Table 1.

**Table 1:** Tested assembly and variants of experimental set-up.

Compensation components	L	23 μH
	C	3.3 nF
Variable load	R_trim_	0–20 Ω
On-board electronics (Secondary side of WPT system)	Diode bridge	H-bridge SiC
	Diode Bridge + DCDC	L_DCDC_ = 10 μH
	I_type_ Bridge	2*L 10 μH, SiC
	U_type_ Bridge	CMM = 900 μH, SiC
Off-board electronics (Primary side of WPT system)	Inverter	H-bridge SiC
	Power supply	Universal
Electrical parameters	Voltage range	Up to 312 V
	Current range	Up to 15 A
	Power limit	500 W
	Frequency	577 kHz

### Backround theory of the impedance matching for SS WPT system considering topology variations for secondary side rectifier

**Figure 2:**
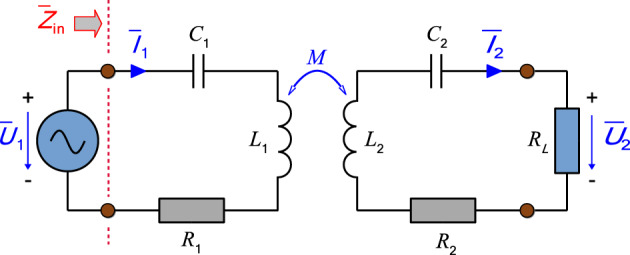
Equivalent circuit of the WPT system using series-series compensation.

Several key conclusions can be derived from the theory of coupling elements of a WPT system using series-series configuration of the compensation network (Fig. 2). First of all, it is about defining the basic circuit description (Eq. (1)) and consequently identify the optimal value of the load resistance (Eqs. (2), (3)), i.e. targeting maximum system efficiency^22,23^.

1 $$\begin{array}{c}-j\frac{1}{\omega {C}_{1}}{\overline{I} }_{s1}+j\omega {L}_{1}{\overline{I} }_{s1}-jM\omega {\overline{I} }_{s2}+{R}_{1}{\overline{I} }_{s1}-{\overline{U} }_{1}=0\\ {-j\frac{1}{\omega {C}_{2}}{\overline{I} }_{s2}+j\omega {L}_{2}{\overline{I} }_{s2}-jM\omega {\overline{I} }_{s1}+R}_{2}{\overline{I} }_{s2}+{R}_{2L}{\overline{I} }_{s2}=0\end{array}$$

2 $${R}_{z\upeta max} = \sqrt{{R}_{1}{R}_{2}+ {M}^{2}{\omega }_{0}^{2}}$$

Considering zero I2R losses (2) changes into (3).

3 $${R}_{L\upeta max} = M{\upomega }_{0}$$

The Eq. (3) identifies that once the ideal situation occurs, i.e., the parasitic resistances of the primary and secondary coils are zero, the optimal value of the load resistance is directly proportional to resonant frequency and the value of mutual inductance.

Since it is a resonance coupling, then Eq. (4) must be satisfied. It defines the resonant frequency as the operating frequency of the WPT system.

4 $${f}_{0} = \frac{1}{2\uppi \sqrt{LC}}$$

**Figure 3:**
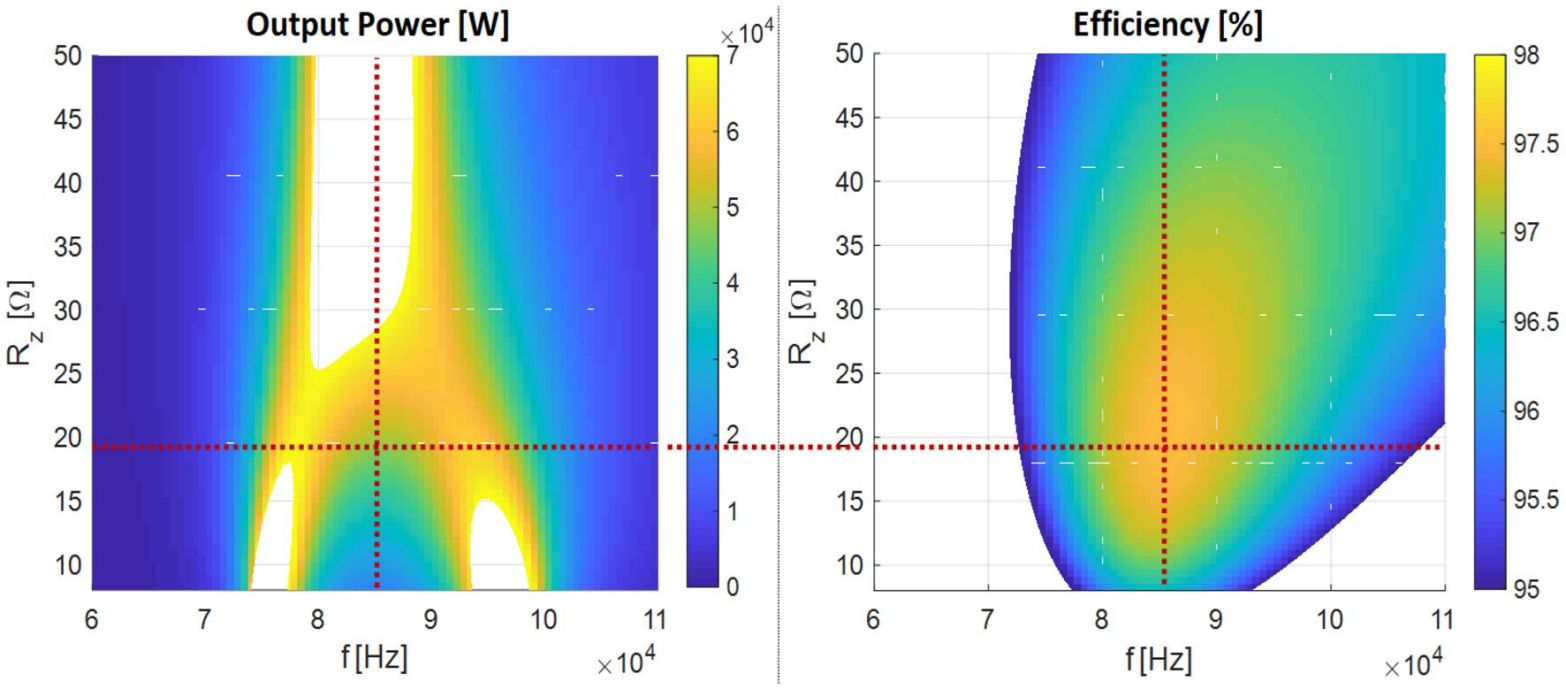
Example of the graphical interpretation of the optimal value of load resistance (65 kW prototype of WPT system), left – dependency of output power, right – dependency of efficiency.

The dependency of the load resistance on the optimal operation point regarding efficiency and output power of the WPT system is graphically interpreted on Fig. 3, which is valid for the 65 kW system power (the highest efficiency (97.5%) refers to the 50 kW of the output power). As it is clear from this figure, if the load of the coupling elements is not impedance-matched, then efficiency decrease would happen, which is an undesirable phenomenon that must be minimized.

As follows from Eq. (2), the optimal load resistance is dependent on the parasitic resistance of the circuit R (R_1_ = R_2_ = R), i.e. due to thermal changes of the ESRL and ESRC parameters. Apart from changes in R, RLηmax also depends on the operating parameter in the form of mutual inductance M, which is a function of the coupling factor between the coupling elements and thus on their relative position (alignment) and distance (transmission distance). A more detailed description regarding coupling elements influences can be found in the literature^1^.

#### Impedance matching of the standard bridge diode rectifier

**Figure 4:**
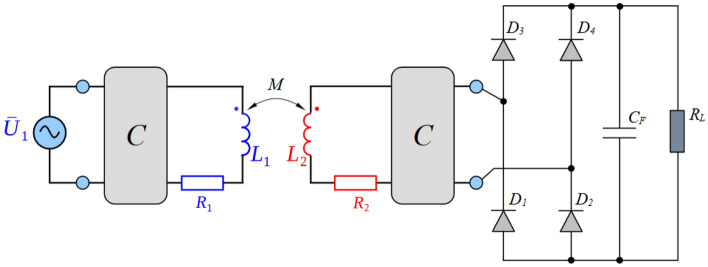
Equivalent schematics of WPT system with current type rectifier.

According to the terms of impedance matching for direct connection of the DC load using the bridge diode rectifier (Fig. 4), the situation can be described by Eq. (5). Impedance conversion is independent of the circuit parameters and is defined only by a physical principle. For the real case, however, Eq. (5) is incomplete, as it does not respect the static and dynamic resistance of individual diodes. Ultimately, the impedance conversion of the diode rectifier will be slightly dependent on the circuit parameters, which can be neglected with minimal error.

Achieving the optimal operating condition of the WPT is therefore possible only by changing the design of the coupling elements, or by changing the resistance of the DC load in order to fulfill the condition of Eq. (3).

5 $${R}_{L\, AC} = \frac{8}{{\uppi }^{2}} {R}_{L DC}$$

#### Impedance matching of the standard bridge diode rectifier with DC/DC converter

Adding a buck DC-DC type of the converter to the diode bridge rectifier, it is possible to achieve an active and continuous impedance matching of the load and compensation element. The impedance conversion is then given by the combination of the rectifier conversion rate (Eq. (5)) and the DC-DC converter (Eq. (6)). The resistance on the DC side of the rectifier R_zDC_ is given by the magnitude of the equivalent resistance of the load R_load eq_, which is given bz the by Ohm's law of the output terminals, by the actual ratio between voltage and absorbed current of the battery, and by the duty cycle value of the DC-DC converter.

The resulting impedance matching function of this combination is given by Eq. (7).

6 $${R}_{L \,DC}= \frac{{R}_{\mathrm{load\, }eqv}}{{D}^{2}} ;D\in \langle 0 ;1\rangle$$

7 $${R}_{L\, AC}=\frac{8}{{{\uppi }^{2} {D}^{2}}^{2}} {R}_{\mathrm{load\, }eqv} ; D\in \langle 0 ;1\rangle$$

According to proper operation of the system, the condition defined by Eq. (8) must be met, i.e. the value of the resistance at the DC side of the rectifier (R_LDC_) must be much higher than the value of the equivalent resistance. The second condition concerns the battery-type load, when the load current cannot exceed the maximum battery current, while the minimum current cannot be lower that the ripple's own value (Eqs. (9), (10)).

8 $${R}_{L \,DC} \ge {R}_{\mathrm{load\, }eqv}$$

9 $${I}_{bat}\epsilon (\langle {I}_{bat\mathrm{ \,min}} ; {I}_{bat{\text{max}}CC}\rangle \wedge \langle {I}_{bat\mathrm{ \,min}} ; \frac{{U}_{bat\, max}}{{R}_{eqv}}\rangle )$$

10 $$\frac{{U}_{DC}}{8 \,n \,{L}_{DC} {f}_{sw\, DCDC}} \ll {I}_{bat \,min}$$

where “n” is representing the transformation ratio of the L_1_ and L_2_ turns (Figs. 1, 3).

#### Impedance matching of the current-doubler diode rectifier

**Figure 5:**
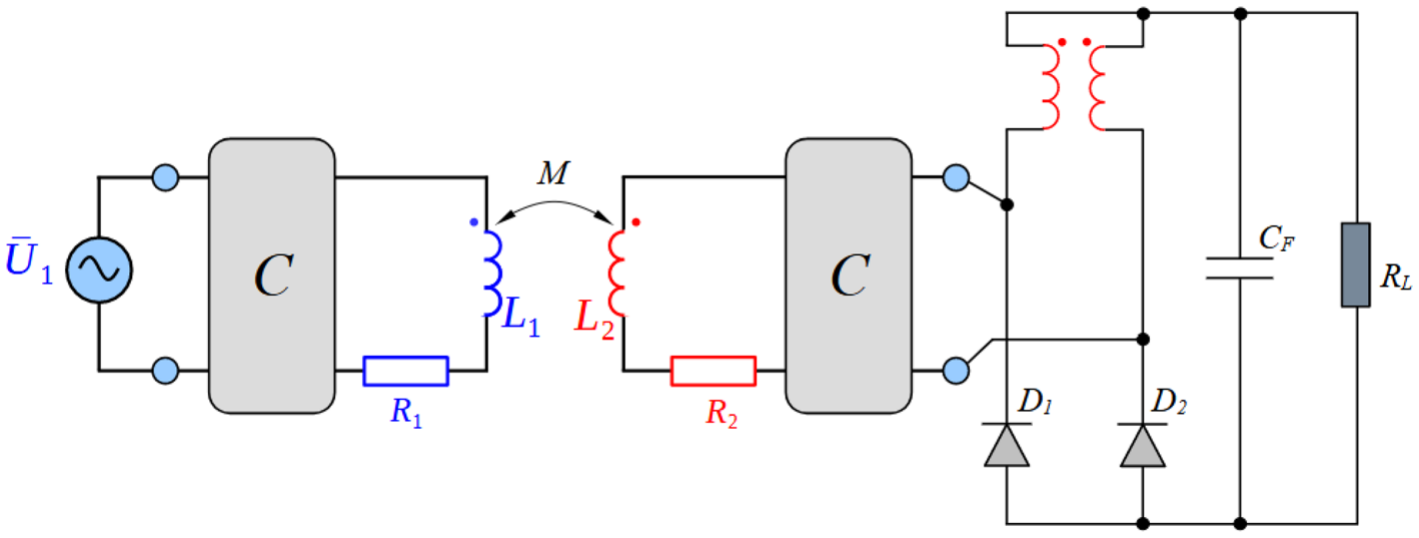
Equivalent schematics of WPT system with current type rectifier.

Equivalent schematic of the WPT system equipped by a special, current-type rectifier with is shown in Fig. 5. Its topology replaces the upper diodes of the standard H-bridge rectifier by inductors, while there is no mutual coupling between them. The value of the inductance to meet operational conditions characterized by the system´s best efficiency performance is given by Eq. (11). So, as is clear, the size of the inductances of I_type RECT_ (current type rectifier) is dependent on the size of the load and thus the condition from Eq. (3) is fulfilled only for one specific value of the load for which the L_I_RECT_ (11) and the coupling elements are designed.

11 $${L}_{I\_RECT}=\frac{{R}_{load \,eqv}}{{\omega }_{0}} \frac{\left(\pi -1\right)}{\pi }$$

The impedance matching ratio for the I_type_ rectifier is defined by Eq. (12), while it is seen that main specific according to the operation of this rectifier type is impedance increasing function.

12 $${R}_{zL \,AC}=\frac{{\uppi }^{2}}{2} {R}_{\mathrm{load\, }eqv}$$

#### Impedance matching of the voltage-doubler diode rectifier

The last alternative considers the second variant of a special type of rectifier with voltage output. The upper diodes of the H-bridge topology are replaced with magnetically coupled inductances in the "common mode" connection according to Fig. 1. Unlike I_type_ RECT, the size of the inductances can be designed for the wide range of values. L_U_RECT_ is limited by a minimum value so that discontinuous character do not occur during the operation with the lowest load current. The U_type_ RECT impedance conversion ratio is given by Eq. (13), which results in a clear increasing impedance function, but approximately half that of the standard U_type_ H-bridge rectifier (Supplementary Information).

13 $${R}_{L \,AC}\approx \frac{32}{{\uppi }^{2}} {R}_{\mathrm{load\, }eqv}$$

## Results

**Figure 6:**
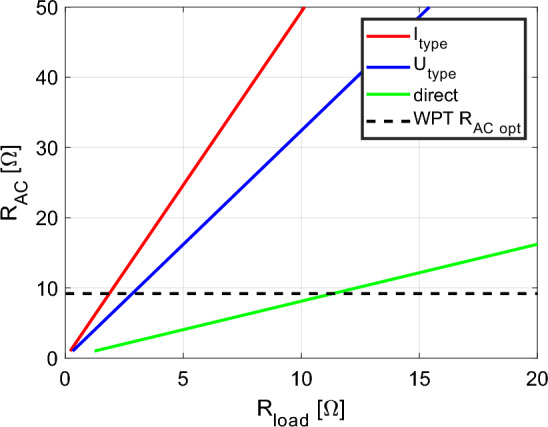
Impedance matching values of individual secondary side rectifiers.

From Eqs. (5), (6), (7), (8), (9), (10), (11), (12) and (13) is seen that the value of the impedance matching in relation to the optimal load (3) is specific for individual variants of secondary side rectifiers discussed in previous part of the paper. Figure 6 shows comparison of the impedance matching values of discussed rectifier types and its relationship to the optimal value of the R_AC_. From this figure is seen, what is the optimal value of the load resistance considering individual secondary side types of rectification.

Table 2 is listing these values for each type. At this point it must be noted that DC-DC connection on the secondary side can adapt the optimal value of R_AC_ due to the possibility of the regulation of the output voltage and/or current.

**Table 2:** Comparison of the values of optimal load resistance for individual secondary side rectifiers.

Variant	R_load opt_
Direct	11.5
DCDC	–
I_type_	1.8
U_type_	2.8

### Time-domain simulation

**Figure 7:**
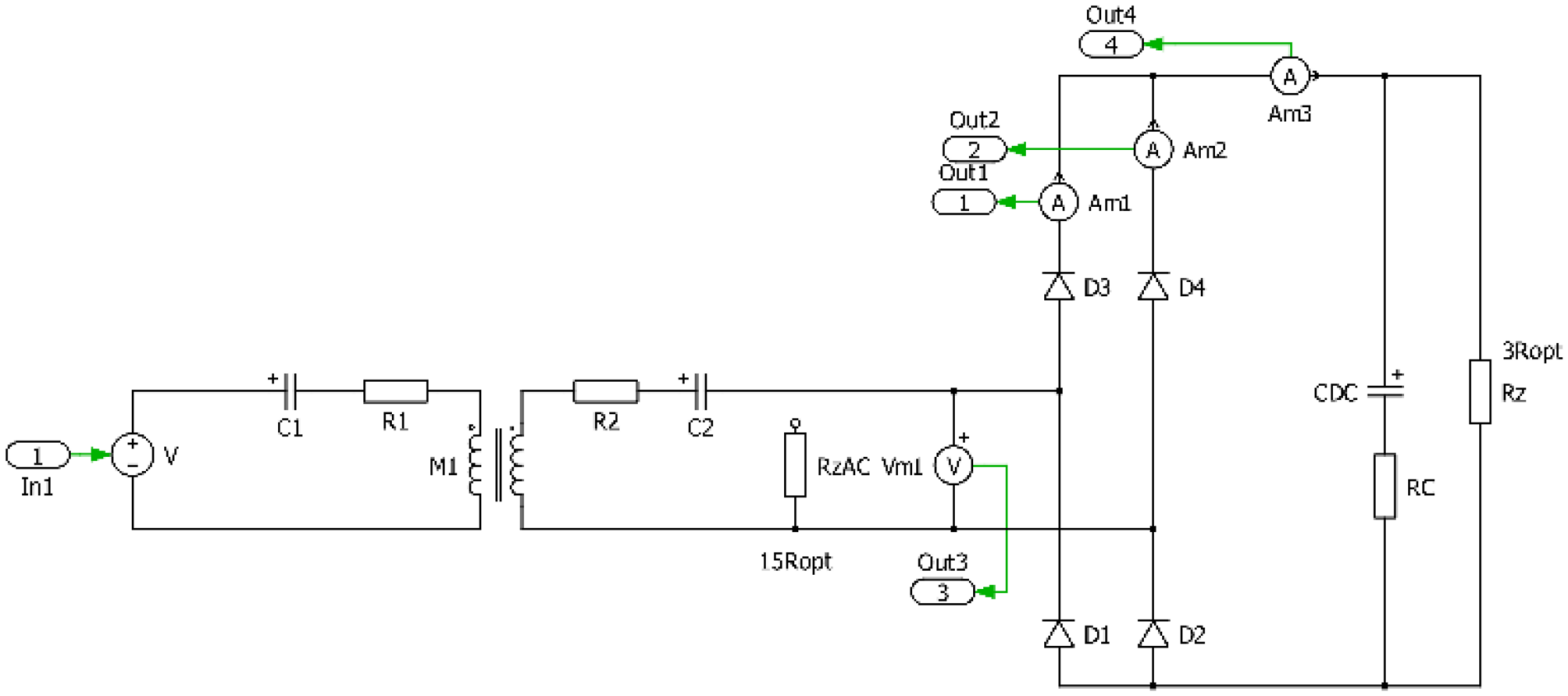
Simulation model of direct connection type.

**Figure 8:**
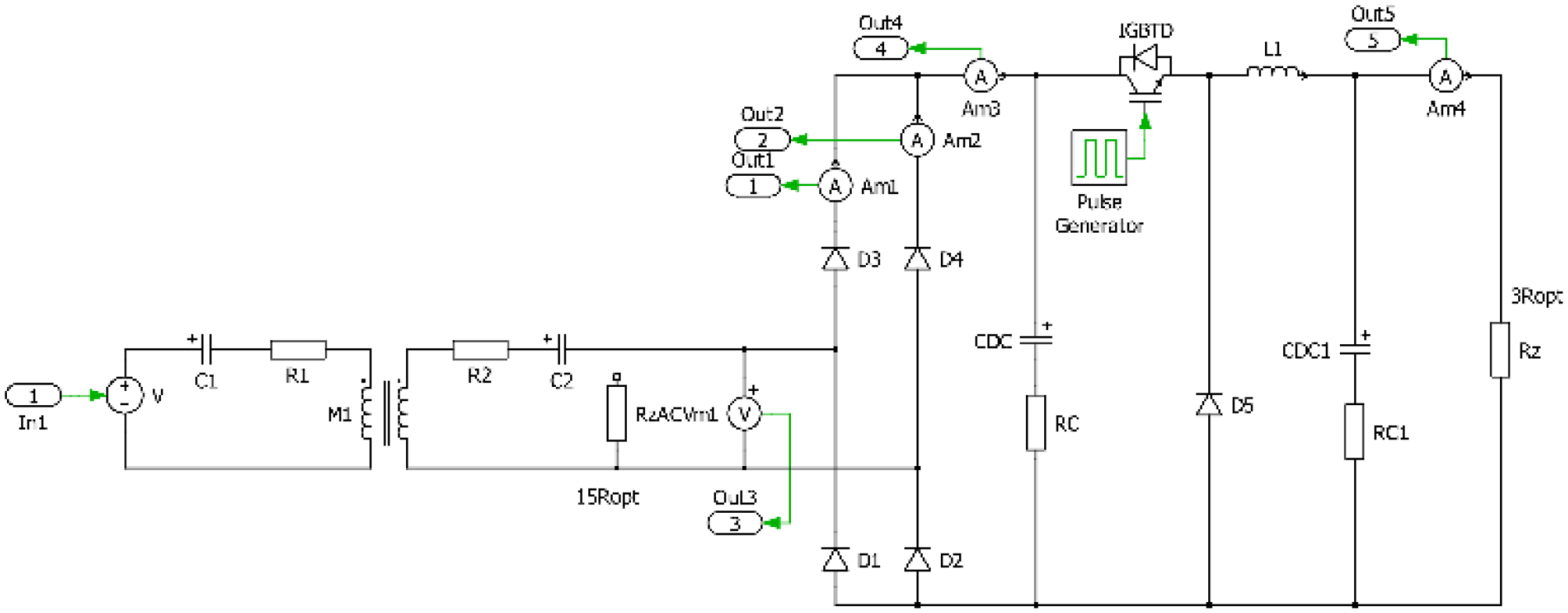
Simulation model of direct connection type with DCDCregulation.

**Figure 9:**
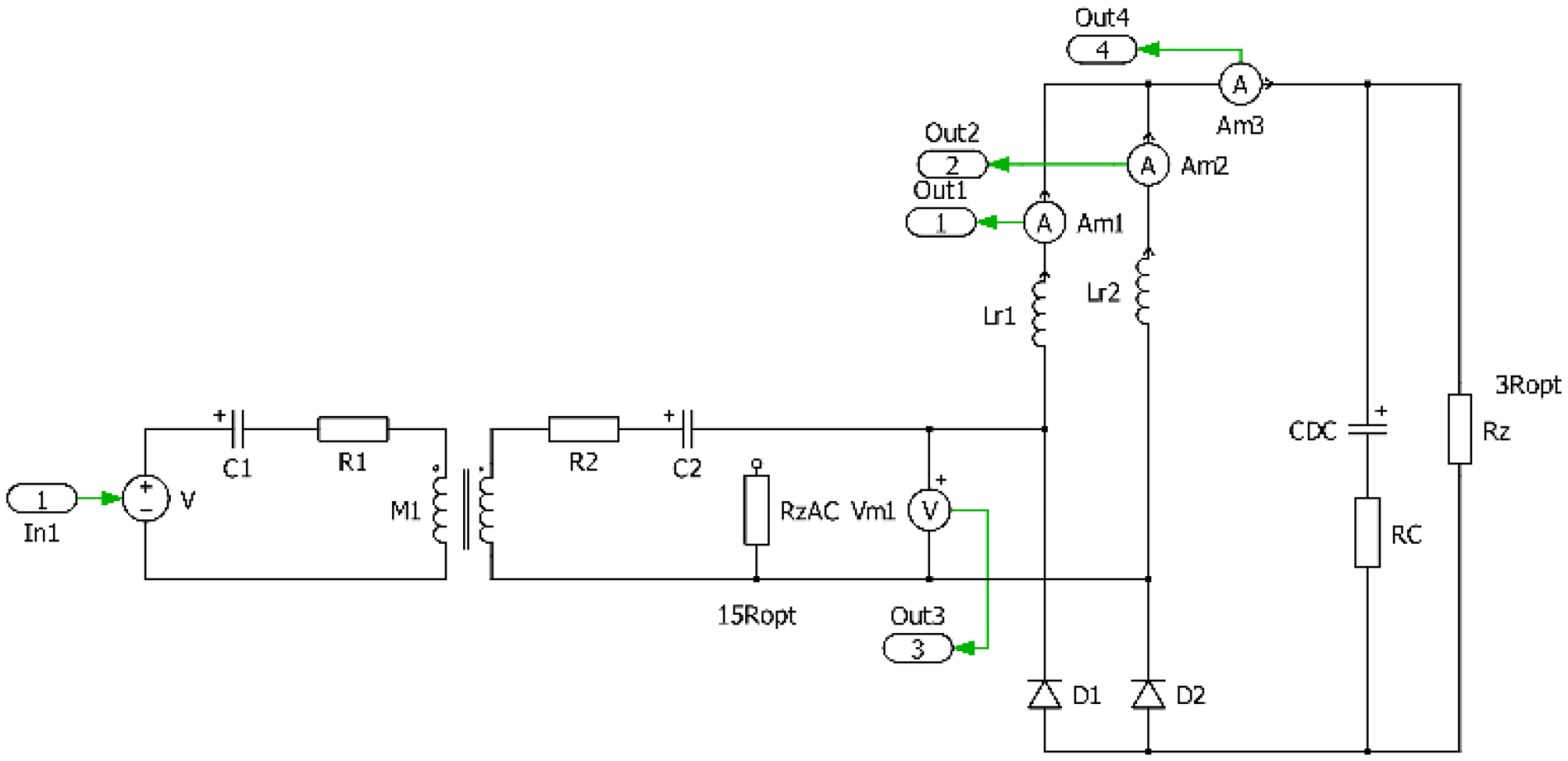
Simulation model of I_type_.

**Figure 10:**
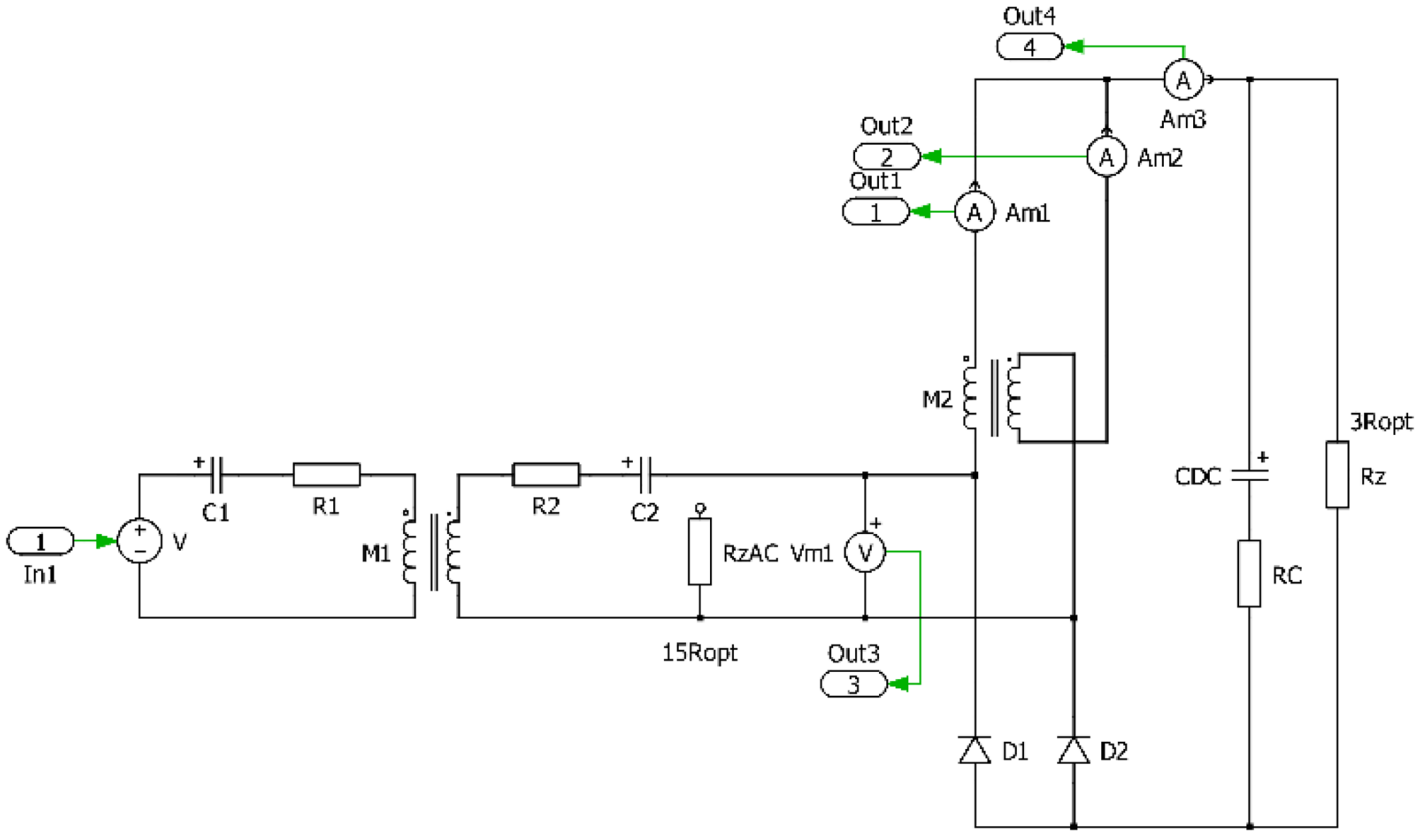
Simulation model of U_type_.

In order to be able to realize the physical prototypes and to provide experimental measurements, first the simulation models in time domain have been realized. Figures 7, 8, 9 and 10 are showing circuit diagrams of considered secondary side rectifiers. The primary side of the WPT system is replaced by the voltage-sourced equivalent circuit, representing the functionality of the primary side inverter. All circuit parameters are defined in Table 1. Regarding U_type_ rectifier, the common mode inductors with very high magnetic coupling must be considered (k → 1).

**Figure 11:**
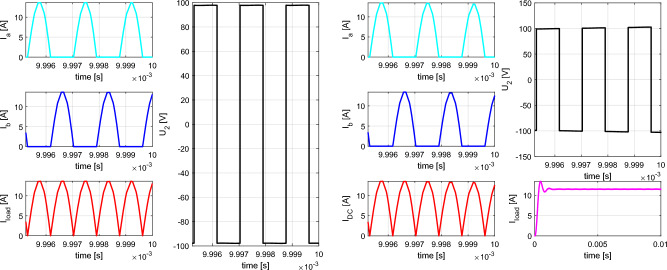
Time-waveforms from simulation models for direct connection (left) and DCDC (right).

**Figure 12:**
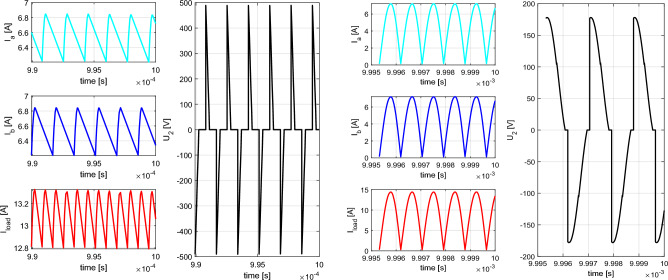
Time-waveforms from simulation models for I_type_ rectifier (left) and U_type_ rectifier (right).

The simulation results shown on Figs. 11 and 12 are representing leg currents of the secondary side rectifier (I_a_, I_b_), load current (I_load_), the current from rectifier to DCDC converter (I_dcdc_) and the voltage on the secondary/receiving coil of the WPT system (U_2_). Individual waveforms are represented for the nominal—steady state operation of the system.

In the case of direct load connection (Fig. 11-left), the presented waveforms are referring to the common operation of diode-bridge rectifier. The currents I_a_ and I_b_ are forming the pulsation of the load current I_load_. The voltage U_2_ has naturally rectangular shape, what is caused by the operational principle of the diode rectifier and with capacitive filter on the DC side. At the same time, for the presented case R_load_ = R_load opt_, U_1m_ = U_2m_ and I_1m_ = I_2m_ apply here.

In the case of connecting a load via a combination of a diode rectifier and a DCDC converter (Fig. 11– right), which has the responsibility for resistance variation, the presented waveforms are similar to the situation when direct connection is considered (R_load_ = 6.37 Ω, duty-cycle = 0.75).

For the situation, when I_type_ rectifier is considered, a phase shift of 90° is visible between the currents I_a_ and I_b_. I_load_ is formed by these two leg currents, while the current ripple is less compared to direct connection. Due to the triangular character of the current, a very unfavorable waveform of the voltage is visible, if transmitting and receiving coil are under consideration. This time waveform of U_2_ (Fig. 12—left) is typical for power supplies which behave as a very stiff current source. It is seen that at the moment of diode commutation, extreme rise of voltage exists, while during the gradual increase of the I_load_ the gradual decrease of U_2_ applies. After this transient the U_2_ voltage is practically zero. The amplitude of the U_2_ pulse is given by Eq. (14).

14 $${{\text{U}}}_{2m}= L\frac{{I}_{2rms}}{tc}=L\frac{\frac{{I}_{load}}{2\sqrt{2}}\frac{{\pi }^{2}}{2}}{{L}_{itype}\frac{{di}_{load}}{{R}_{load}{I}_{load}}}= L\frac{\frac{{I}_{load}}{2\sqrt{2}}\frac{{\pi }^{2}}{2}}{{L}_{itype}\frac{\frac{1}{{2f}_{0}}\frac{{I}_{load}{R}_{load}}{{L}_{itype}}}{{R}_{load}{I}_{load}}}= L\frac{\frac{{I}_{load}}{2\sqrt{2}}\frac{{\pi }^{2}}{2}}{\frac{1}{2{f}_{0}}}= L\frac{{\pi }^{2}{f}_{0}{I}_{load}}{2\sqrt{2}}$$

In the case of the variant with the U_type_ rectifier, a phase-shift is 0° between the currents I_a_ and I_b_. Similarly to previous situation, I_load_ is formed by the leg currents. Depending on the quality (coupling factor) of the common-mode inductor, the currents are half-wave in nature, which has a positive impact on the U_2_ voltage waveform. After reaching right the I_load_ effective value, U_2_ begins to fall (Fig. 12—right). The amplitude of the U_2_ pulse is given by Eq. (15).

15 $${{\text{U}}}_{2m}=L\frac{{I}_{2rms}}{tc}= \frac{\frac{{I}_{load\_m}}{\sqrt{2}}}{\frac{1}{2{f}_{0}}}=2L{f}_{0}{I}_{load}$$

**Figure 13:**
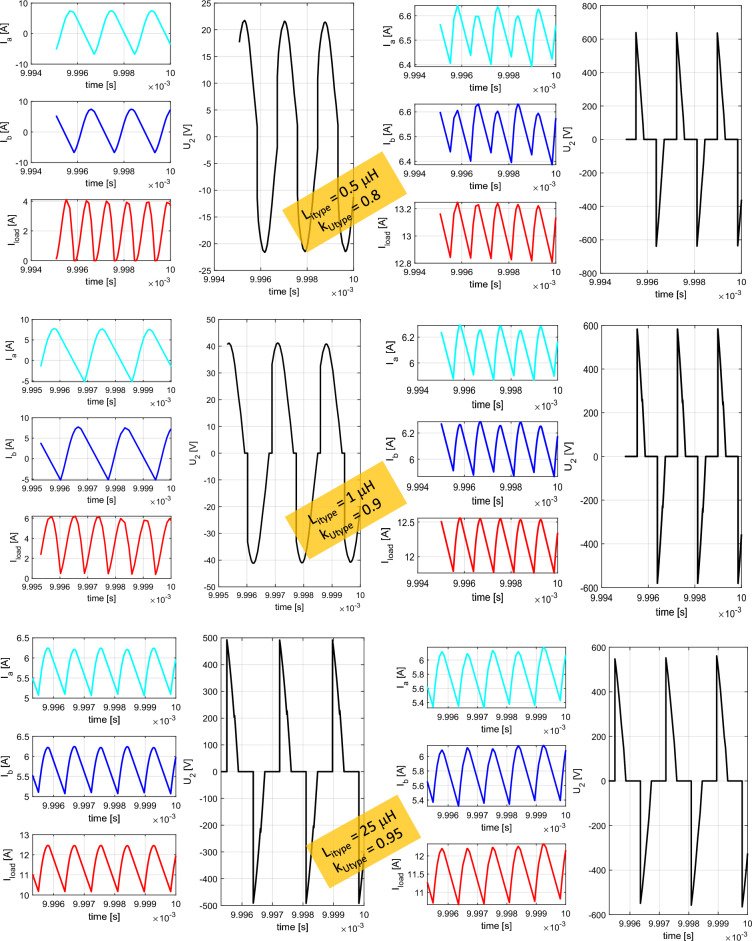

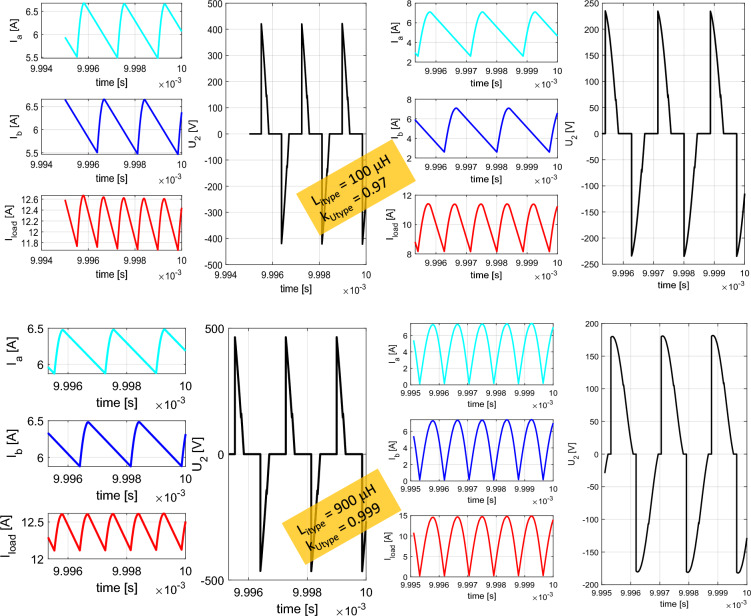
Time-waveforms from simulation models for I_type_ rectifier (left) and U_type_ rectifier (right) during parametric change of the inductance values (I_type_) and coupling factor (U_type_).

From the previous simulation analysis is seen, that the unfavorable effect for U_type_ and I_type_ rectifier is reflected within the time-waveform of the voltage on coupling coils of WPT system. Both types use inductors within the main circuit. It is therefore desirable to investigate the influence of the values of inductances on the operational behavior of I_type_ and U_type_ rectifiers. For the case of U_type_ rectifier, the variable parameter is coupling factor of the common mode choke (from 0.8 to 0.999). I_type_ rectifier has variation within the value of the inductance of both inductors within the range 1 µH–900 µH. Figure 13 is showing time-waveforms for individual situations.

As a result of this analysis, next assumption can be made:

- Reduction of the value of inductances of I_type_ rectifier inductors, below the value, whish is determined by Eq. (11) leads to decrease of the average value of rectifier leg´s currents resulting in generation of circulation currents. This causes reduction of the commutation voltage, efficiency and the amount of the transferred power.
- In the case of U_type_ rectifier, the reduction of the coupling factor below 0.97 leads to gradual transition from U_type_ rectifier to I_type_ rectifier. At first, the commutation voltage only increases, which later saturates and the average value of the currents begins to decrease rapidly.

### Experimental measurements

**Figure 14:**
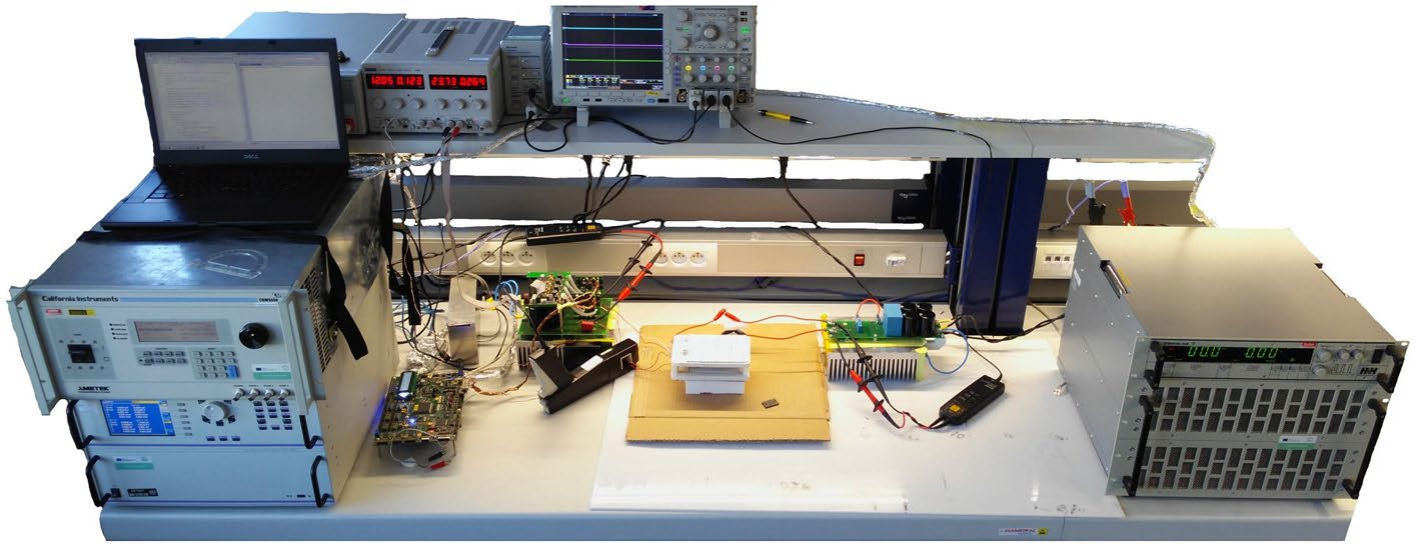
Experimental set-up for evaluation of the properties of secondary side rectifiers of the WPT system (coupling coils are in the middle of the experimental set-up).

**Figure 15:**
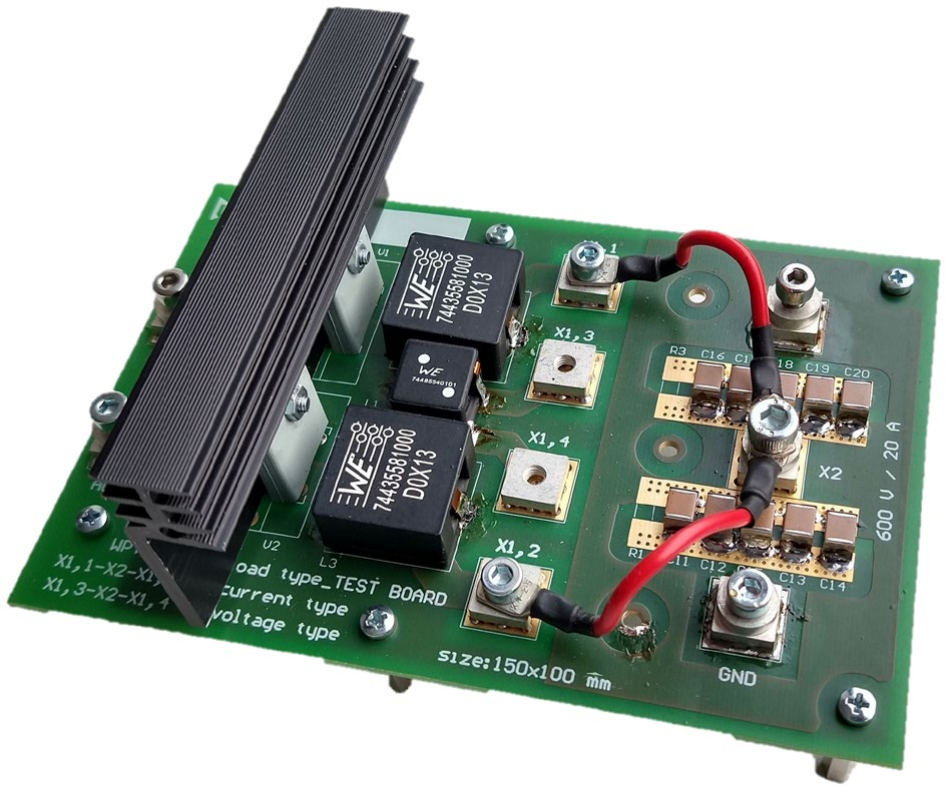
Reconfigurable prototype of secondary side rectifier (boar contains all discussed topologies).

The aim of the experimental measurements is to verify the properties of individual rectifier types, while efficiency performance of whole WPT system will be evaluated. The experimental set-up is shown in Fig. 14. Table 3 shows the input–output parameter during measurements. Figure 15 is representing the prototype of reconfigurable topology of secondary side rectifier, i.e. PCB enables to configure all of discussed types of rectifier.

**Table 3:** Operational parameters, component values and instrumentation list of designed WPT system.

Input voltage (V)	Up to 312 V, based on battery voltage	
Regulated current (A)	Up to 15 A	
Switching frequency (kHz)	577 kHz	
Output power (W)	Up to 500 W	
Coupling elements values	L_1_ = L_2_ = 23 μH	H-bridge SiC
	C_1_ = C_2_ = 3,3 nF	Universal
Rectifiers components	Diode rectifier	H-bridge SiC
	H-bridge SiC + DCDC	L_DCDC_ = 10 μH
	I_type_ bridge	2*L of 10 μH, SiC
	U_type_ bridge	CMM = 900 μH, SiC
Power source	California instruments	CSW 5550
Power analyzes	ZES zimmer	LMG 500
Electronic load	H&H	ZS 7080
Oscilloscope	Tektronix	MSO 4104B
Control board	RICE	Include TMS320F28335
Laboratory source	TTI	EX354 RT

The measurements were processed automatically, i.e. with the help of the supervisor control system (PC), and after the setting of the measuring algorithm, individual devices were automatically controlled, e.g. electronic load, where the load of the WPT system changed in defined steps. Subsequently, the measured data were automatically uploaded to the computer, where graphical visualization have been performed.

**Figure 16:**
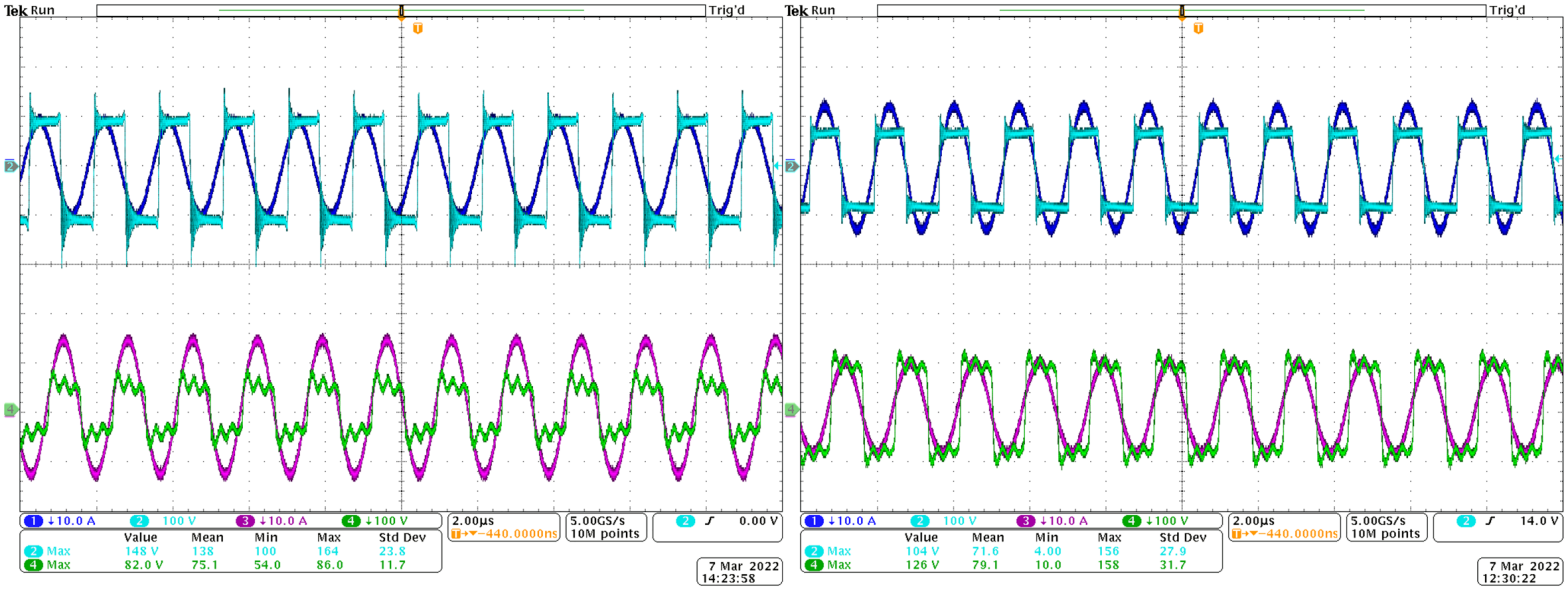
Time-waveforms from the experimental measurement during steady-state of operation at *R*_*load*_ = 5 Ω, left—direct connection, right—DCDC connection.

**Figure 17:**
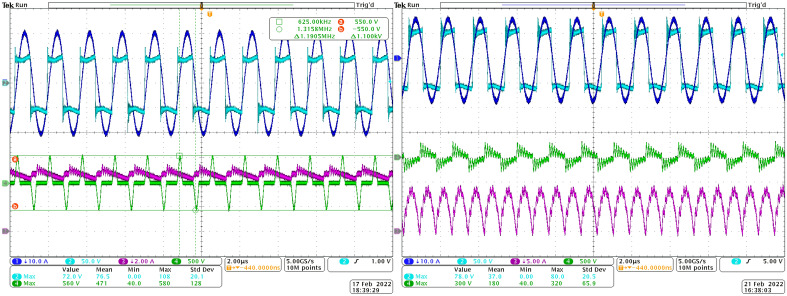
Time-waveforms from the experimental measurement during steady-state of operation at *R*_*load*_ = 5 Ω, left—I_type_ connection, right—U_type_ connection.

**Figure 18:**
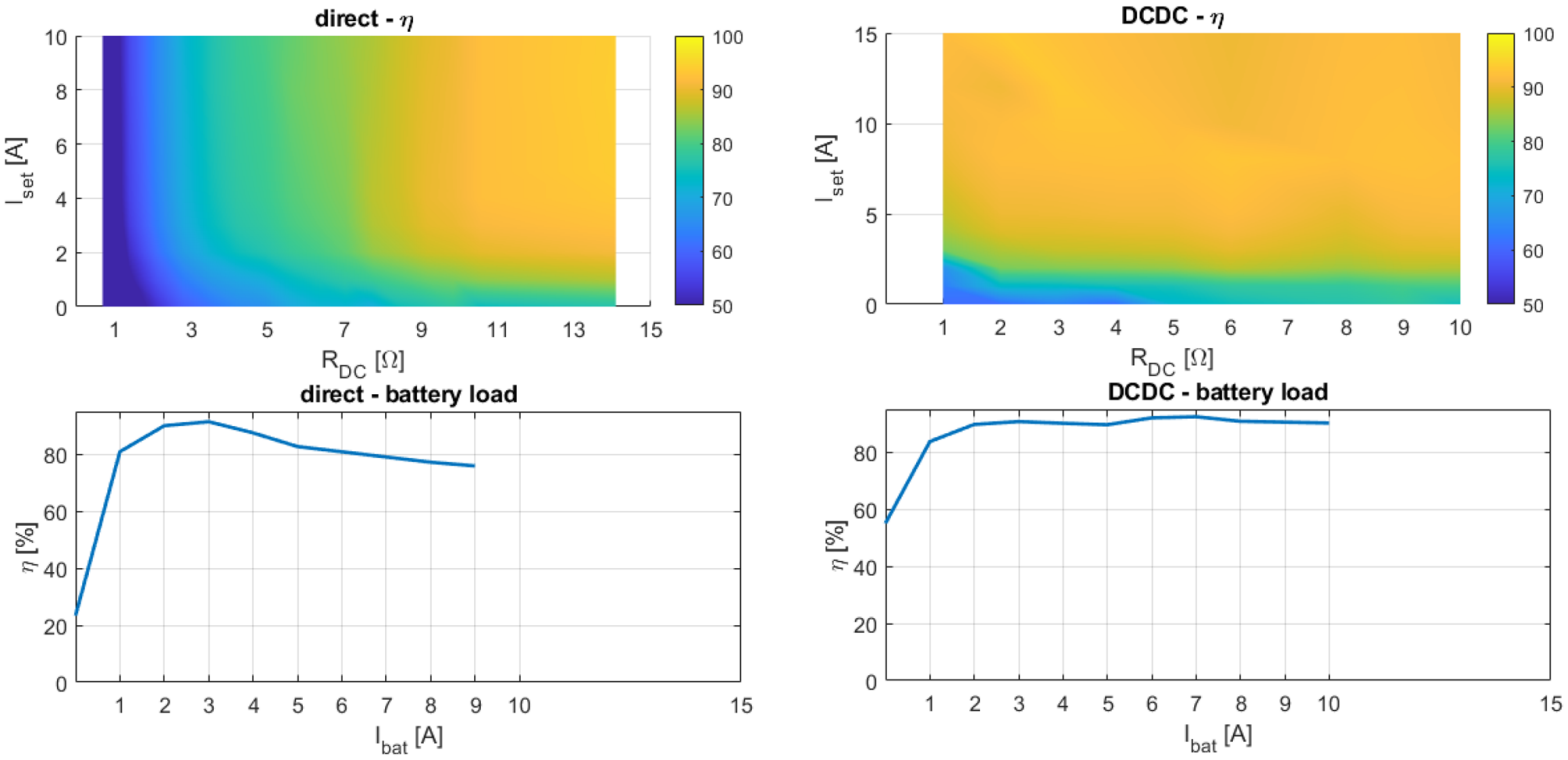
Character maps from experimental measurements of direct rectifier connection (left) and DCDC connection (right).

**Figure 19:**
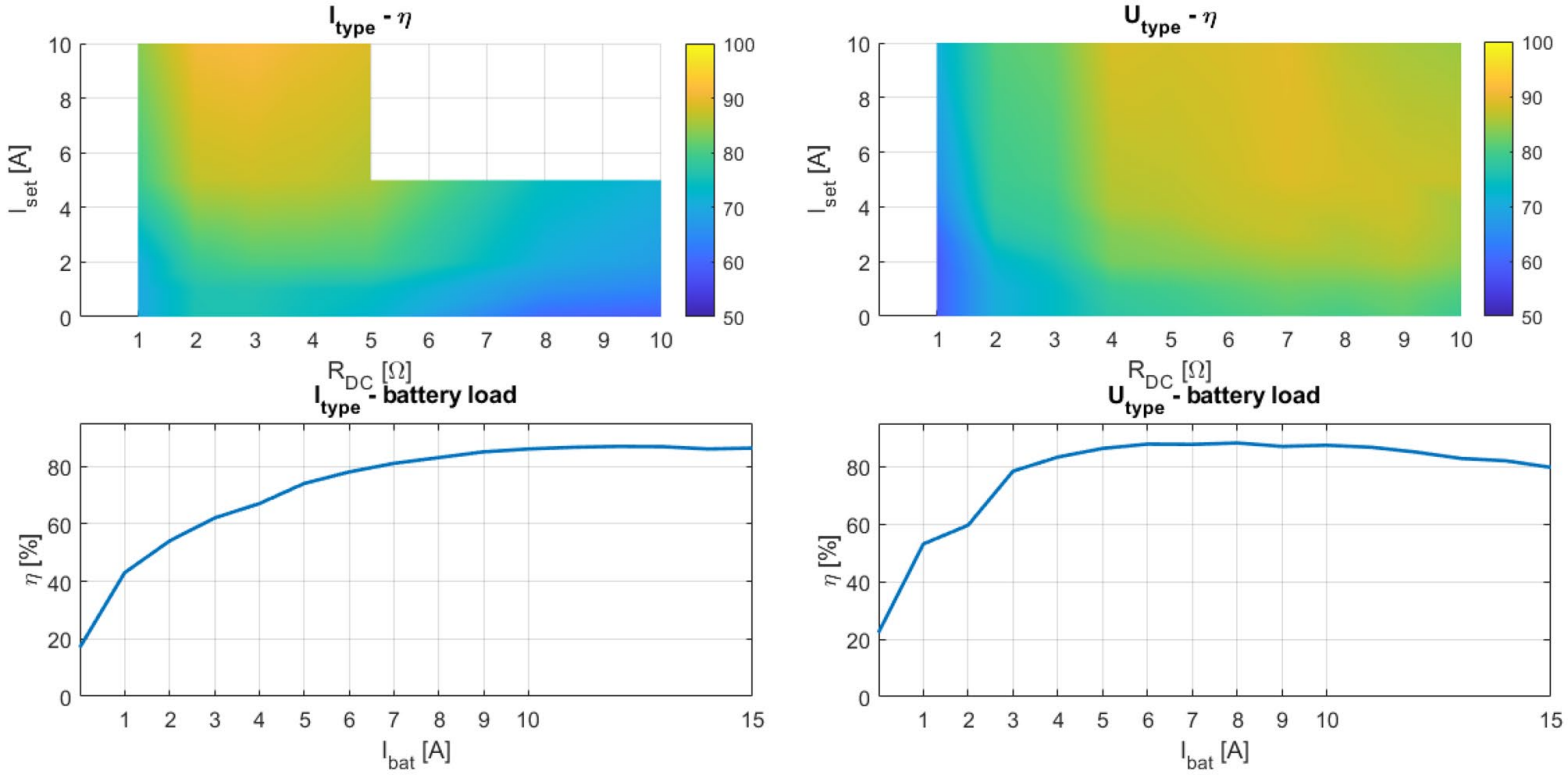
Character maps from experimental measurements of I_type_ rectifier connection (left) and U_type_ rectifier connection.

To confirm results from the simulation analyses, the measurement of time-waveforms was performed, while the influence of the type of rectifier was validated. Set *R*_*load*_ is the same for all the cases of Figs. 16 and 17 to imagine rectifier influence better, especially in connection with Figs. 18 and 19 and chapter 3.2. Recorded variables are:

- Voltage on the transmitting coil—light blue waveform;
- Current of the transmitting coil—dark blue waveform;
- Voltage on the receiving coil—green waveform;
- Current on the receiving coil/leg of the rectifier—pink waveform.

The goal of this investigation was to proof the time-waveforms of the simulation analysis, while focus is given on the voltage amplitudes on the secondary side receiving coil, as well as the current of the rectifier leg. Table 4 is listing the values on the individual components. It is seen that the simulation expectations are confirmed, and the differences between the measurement and simulation are very low (considering value of the rectifier leg current the relative error is approximately 7.7% for I_type_ and 5% for U_type_).

**Table 4:** Comparison of the values of the voltage and current on I_type_ and U_type_ rectifier.

Rectifier variant	U_2_ (Vp-p)		I_2_ (Ap-p)	
	Simulation	Measurement	Simulation	Measurement
I_type_	500	500	13	12
U_type_	180	250 (180 without oscillations)	10.6	10.039

Experimental verification is done for all four connections under these test conditions: DC load form 1 by 1 to 10/15 Ω and DC load current from 1 by 1 to 10/15 A (ideally). See Figs. 18 and 19.

The results from the experimental verification are presented in three ways. First, they are represented as efficiency map, next as the load current map and finally as the efficiency dependency on the load current in case of *R*_*DC*_ optimum for each WPT to battery connection case. The load current is representing eventual battery current, therefore electronic load was operated equally to the operation of battery charging.

### Discussion to experimental results

From Fig. 18 left is seen, that direct connection between coupling element and rectifier is advantageous only for the applications, where almost constant load is expected. For this specific value of load, the coupling elements are designed adequately. Alternative with DCDC converter between load and coupling element has the possibility to match the impedance for the whole range of the load values and power variations. The additional losses caused by high currents are compensated as well (Fig. 18—left and right bottom part). So, connection using DCDC converter is suitable for wide range of currents opposite to the others. The only limitation would be a small value of currents if low load resistance is considered camming from pulse converter operation background.

The I_type_ connection of the rectifier is suitable for very small values of load resistances, i.e., for high values of secondary side currents (Fig. 19 left). The area of the effective utilization of this configuration is slightly narrow compared to the other options, so the application with variable load is not preferable. The last configuration of the rectifier, U_type_ is applicable for the systems with higher resistive load. The effective area of its utilization is wider compared to the I_type_ rectifier; thus the use is preferable also for the applications with slightly variable load resistance. Bottom parts of Fig. 19 shows efficiency dependence on load current at case of optimal *R*_*DC*_ too. Finally, I_type_ is more beneficial for the highest load currents and U_type_ for the medium load currents.

## Conclusions

The main objective of this research was to investigate the possibilities of alternative solutions of the on-board electronics of the wireless power charging system. We are discussing here about the secondary side rectification of WPT system, whereby focus was given on four circuit topologies. The investigated variants were standard bridge diode rectifier, standard bridge diode rectifier supplemented by DC/DC converter at the output, modified diode bridge rectifier to current type (I_type_) and modified diode bridge rectifier to voltage type (U_type_). It was discovered that each of the rectifier has different properties related to the identification of optimal value of the load resistance of WPT. In other words, the possibilities for impedance matching adjustments have been identified. From the simulation and experimental results, the conclusion can be summarized that the derived variants of the bridge rectifiers, i.e. I_type_ and U_type_ are equivalent, but in detail the variant with a voltage output (with a common-mode inductor) is more suitable. It is simpler to design, takes up less space, does not exhibit large commutation overvoltage and has a voltage output. In the case of high-quality design and determination of load change limits, the variants are effectively comparable to the rectifier variant equipped by DC/DC converter. In general, I_type_ and U_type_ rectifier variants are more suitable for a fixed or slightly changing load than for a big battery charging, where CV/CC charging profile has significantly impact to the battery input equivalent load. Once the battery charging is considered, the most suitable option is the use of active impedance adaptation, i.e. the utilization of secondary side rectifier with DC/DC converter, when multiple times higher efficiency and its stability are achieved throughout the entire charging in the CC–CV cycle compared to other investigated topologies. The only disadvantage here can be operate within the region with small values of the charging currents, when DC/DC converter is forced to the discontinuous mode of operation resulting in the reduction of the system efficiency. However, this can be largely eliminated with appropriate design.

Proposed I_type_ rectifier is suitable for low loads with high currents. For example, CC battery or supercapacitor charging in electronic and smaoll drives (e.g. micro drilling, actuators) applications. Proposed U_type_ rectifier is suitable for medium current application with slightly higher input impedance e.g., electronic application supplying, backup or small batteries charging small actuators supplying act. In contrary to the connection using DCDC converter—Big advantage of I_type_ and U_type_ rectifier is fully passive, cheap and small solution of impedance matching for a specifics applications with a small fluctuation of input equivalent impedance.

Partial results of this research also show a certain feature of the derived topologies of the diode rectifier, for example enabling the transfer of Joule losses from the primary side of the WPT system to the secondary side. This ability was also proven by measurements, thus for special applications, the U_type_ rectifier may be a more suitable choice (airtight applications, etc).

### Supplementary Information

Supplementary Information.

## Data Availability

All data generated or analysed during this study are included in this published article.
