# Supplementary material for: Optimization of series-series compensated wireless power transfer system using alternative secondary side rectification
Source: Sci Rep. 2024 Jan 12;14:1191. doi: 10.1038/s41598-023-49305-9 (PMC10786924; doi:10.1038/s41598-023-49305-9)
Supplement: Supplementary file 1 — Supplementary Information. [file 41598_2023_49305_MOESM1_ESM.docx]

Appendix – The example of the rectifier analysis targeting derivation of (11) and (12)

The derivation is based on the equivalent scheme show in Figure A1.


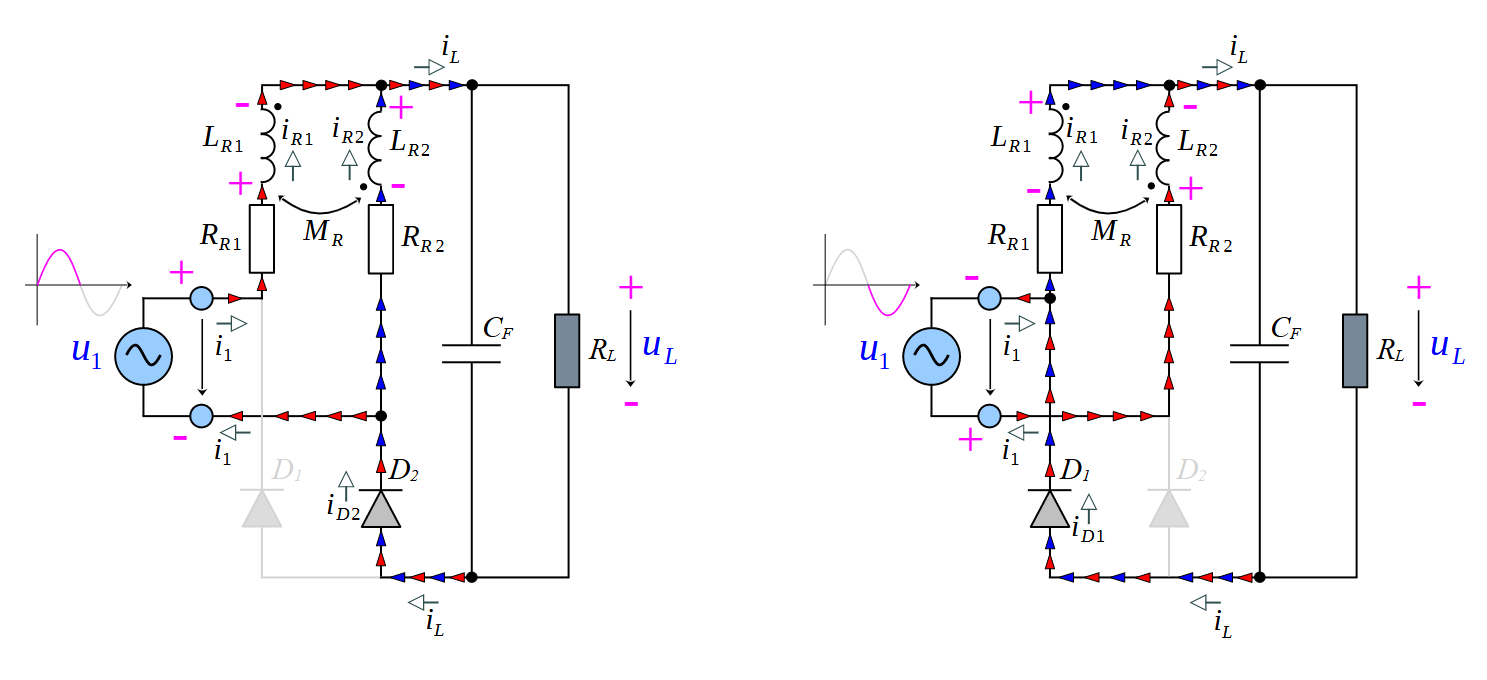


**Figure A1.**  Rectifier operating modes; "A" mode on the left, "B" mode on the right.

Both windings have identical parameters due to symmetry. Assuming a sinusoidal input voltage $u_{1}$, we can divide the duty cycle of the rectifier into two symmetrical modes (mode A and mode B here).

In the mode „A“ ($0<t<T/2$), the input voltage $u_{1}$ has a positive value. A current is taken from the source, which flows through the inductance $L_{R1}$, the load $\left. C_{F} \right\|R_{L}$ and returns to the source through the diode $D_{2}$. At the same time, the energy of $L_{R2}$ is discharged in the form of current closing through the load and diode $D_{2}$.

In the mode „B“ ($T/2<t<T$), the input voltage $u_{1}$ has a negative value. A current is taken from the source, which flows through the inductance $L_{R2}$, the load $\left. C_{F} \right\|R_{L}$ and returns to the source through the diode $D_{1}$. At the same time, the energy of $L_{R2}$ is discharged in the form of current closing through the load and diode $D_{1}$.

We can write the equation of state for both modes (A1).

| $\left( 0<t<\frac{T}{2} \right)\left\{ \begin{aligned} \frac{d}{dt}i_{R1}=\frac{1}{L_{R1}}\left( u_{1}-u_{L}-u_{D2}-R_{R1}i_{R1} \right) \\ \frac{d}{dt}i_{R2}=\frac{1}{L_{R2}}\left( u_{L}+u_{D1}+R_{R2}i_{R2} \right) \end{aligned} \right.$  $\left( \frac{T}{2}<t<T \right)\left\{ \begin{aligned} \frac{d}{dt}i_{R2}=\frac{1}{L_{R2}}\left( u_{1}-u_{L}-u_{D1}-R_{R2}i_{R2} \right) \\ \frac{d}{dt}i_{R1}=\frac{1}{L_{R1}}\left( u_{L}+u_{D2}+R_{R1}i_{R1} \right) \end{aligned} \right.$ | (A1) |
| --- | --- |

Considering $R_{R1}=R_{R2}=0$ and diodes having zero threshold voltage $u_{D1}=u_{D2}=0$, we obtain (A2).

| $\left( 0<t<\frac{T}{2} \right)\left\{ \begin{aligned} \frac{d}{dt}i_{R1}=\frac{1}{L_{R1}}\left( u_{1}-u_{L} \right) \\ \frac{d}{dt}i_{R2}=\frac{u_{L}}{L_{R2}} \end{aligned} \right.$  $\left( \frac{T}{2}<t<T \right)\left\{ \begin{aligned} \frac{d}{dt}i_{R2}=\frac{1}{L_{R2}}\left( u_{1}-u_{L} \right) \\ \frac{d}{dt}i_{R1}=\frac{u_{L}}{L_{R1}} \end{aligned} \right.$ | (A2) |
| --- | --- |

If $L_{R1}=L_{R2}$, then we can find similarities of $i_{R1}$ a $i_{R2}$. Integrating (A2), we get (A3) and (A4).

| $\left( 0<t<\frac{T}{2} \right):i_{R1}\left( t \right)=i_{R1}\left( 0 \right)+\frac{U_{m}}{\omega L_{R1}}\left( 1-cos\left( \omega t \right) \right)-\frac{u_{L}}{L_{R1}}t$  $\left( \frac{T}{2}<t<T \right):i_{R1}\left( t \right)=i_{R1}\left( \frac{T}{2} \right)+\frac{u_{Z}}{L_{R1}}\left( \frac{T}{2}-t \right)$ | (A3) |
| --- | --- |

| $\left( 0<t<\frac{T}{2} \right):i_{R2}\left( t \right)=i_{R2}\left( 0 \right)-\frac{u_{Z}}{L_{R2}}t$  $\left( \frac{T}{2}<t<T \right):i_{R2}\left( t \right)=i_{R2}\left( \frac{T}{2} \right)+\frac{U_{m}}{\omega L_{R2}}\left( 1+cos\left( \omega t \right) \right)+\frac{u_{L}}{L_{R2}}\left( \frac{T}{2}-t \right)$ | (A4) |
| --- | --- |

Relabeling according to $L_{R1}=L_{R2}=L_{R0}$, gives us (A5) and (A6).

| $\left( 0<t<\frac{T}{2} \right):i_{R1}\left( t \right)=i_{R1}\left( 0 \right)+\frac{U_{m}}{\omega L_{R0}}\left( 1-cos\left( \omega t \right) \right)-\frac{u_{L}}{L_{R0}}t$  $\left( \frac{T}{2}<t<T \right):i_{R1}\left( t \right)=i_{R1}\left( \frac{T}{2} \right)+\frac{u_{L}}{L_{R0}}\left( \frac{T}{2}-t \right)$ | (A5) |
| --- | --- |

| $\left( 0<t<\frac{T}{2} \right):i_{R2}\left( t \right)=i_{R2}\left( 0 \right)-\frac{u_{L}}{L_{R0}}t$  $\left( \frac{T}{2}<t<T \right):i_{R2}\left( t \right)=i_{R2}\left( \frac{T}{2} \right)+\frac{U_{m}}{{\omega L}_{R0}}\left( 1+cos\left( \omega t \right) \right)+\frac{u_{L}}{L_{R0}}\left( \frac{T}{2}-t \right)$ | (A6) |
| --- | --- |

Due to the periodicity of both currents, the identities (A7) may be used to find their average values (A8).

| $i_{R2}\left( 0 \right)=i_{R1}\left( \frac{T}{2} \right)$  $i_{R2}\left( \frac{T}{2} \right)=i_{R1}\left( 0 \right)$ | (A7) |
| --- | --- |

| $I_{R1}=\frac{1}{T}\left[ \int_{0}^{\frac{T}{2}} \left( i_{R1}\left( 0 \right)+\frac{U_{m}}{{\omega L}_{R0}}\left( 1-cos\left( \omega t \right) \right)-\frac{u_{L}}{L_{R0}}t \right)dt+\int_{\frac{T}{2}}^{T} \left( i_{R1}\left( 0 \right)+\frac{U_{m}}{{\omega L}_{R0}}\left( 1-cos\left( \omega\frac{T}{2} \right) \right)-\frac{u_{Z}}{L_{R0}}\frac{T}{2}+\frac{u_{Z}}{L_{R0}}\left( \frac{T}{2}-t \right) \right)dt \right]=i_{R1}\left( 0 \right)+\frac{3}{2}\frac{U_{m}}{\omega L_{R0}}-\frac{T}{2}\frac{u_{Z}}{L_{R0}}$  $I_{R2}=\frac{1}{T}\left[ \int_{0}^{\frac{T}{2}} \left( i_{R1}\left( 0 \right)+\frac{U_{m}}{{\omega L}_{R0}}\left( 1-cos\left( \omega t \right) \right)-\frac{u_{L}}{L_{R0}}\frac{T}{2}-\frac{u_{L}}{L_{R0}}t \right)dt+\int_{\frac{T}{2}}^{T} \left( i_{R1}\left( 0 \right)+\frac{U_{m}}{{\omega L}_{R0}}\left( 1+cos\left( \omega\frac{T}{2} \right) \right)+\frac{u_{L}}{L_{R0}}\left( \frac{T}{2}-t \right) \right)dt \right]==i_{R1}\left( 0 \right)+\frac{3}{2}\frac{U_{m}}{\omega L_{R0}}-\frac{T}{2}\frac{u_{L}}{L_{R0}}$ | (A8) |
| --- | --- |

Considering the load voltage as constant and $i_{R1}\left( T \right)=i_{R1}\left( 0 \right)$ we compose (A9),

| $i_{R1}\left( \frac{T}{2} \right)+\frac{U_{L}}{L_{R0}}\left( \frac{T}{2}-T \right)=i_{R1}\left( 0 \right)$ | (A9) |
| --- | --- |

from which the voltage ration can be found as (A10)

| $i_{R1}\left( 0 \right)+2\frac{U_{m}}{\omega L_{R0}}-\frac{U_{L}}{L_{R0}}\frac{T}{2}+\frac{U_{L}}{L_{R0}}\left( \frac{T}{2}-T \right)=i_{R1}\left( 0 \right)$  $⇓$  $2\frac{U_{m}}{2\pi fL_{R0}}=\frac{U_{L}}{L_{R0}}\frac{1}{f}$  $⇓$  $\frac{\sqrt{2}}{\pi}U_{1}=U_{L}$ | (A10) |
| --- | --- |

Considering power invariance, i.e., $U_{1}I_{1}=U_{L}I_{L}$, we derive the current ration of the rectifier (A11).

| $\frac{\pi\sqrt{2}}{2}I_{1}=I_{L}$ | (A11) |
| --- | --- |

The impedance ratio it then (A12).

| $Z_{1}=\frac{U_{1}}{I_{1}}=\frac{U_{L}\frac{\pi}{\sqrt{2}}}{I_{L}\frac{2}{\pi\sqrt{2}}}=Z_{L}\frac{\pi^{2}}{2}$ | (A12) |
| --- | --- |

The current $i_{R1}\left( 0 \right)$, is needed to calculate all the system currents. So, if the load current is given by summation of $i_{R1}\left( t \right)$ and $i_{R2}\left( t \right)$, we write (A13),

| $I_{L}=\frac{U_{L}}{R_{L}}=I_{R1}+I_{R2}=2\left( i_{R1}\left( 0 \right)+\frac{3}{2}\frac{U_{m}}{\omega L_{R0}}-\frac{T}{2}\frac{U_{L}}{L_{R0}} \right)$ | (A13) |
| --- | --- |

which gives (A14).

| $i_{R1}\left( 0 \right)=\frac{U_{L}}{2R_{L}}-\frac{U_{L}\pi}{2\omega L_{R0}}$ | (A14) |
| --- | --- |

Combination (A15)

| $\left( 0<t<\frac{T}{2} \right):i_{1}\left( t \right)=i_{R1}\left( t \right)=i_{R1}\left( 0 \right)+\frac{U_{m}}{\omega L_{R0}}\left( 1-cos\left( \omega t \right) \right)-\frac{U_{L}}{L_{R0}}t$  $\left( \frac{T}{2}<t<T \right):i_{1}\left( t \right)=-i_{R2}\left( t \right)=-\left[ i_{R2}\left( \frac{T}{2} \right)+\frac{U_{m}}{{\omega L}_{R0}}\left( 1+cos\left( \omega t \right) \right)+\frac{U_{L}}{L_{R0}}\left( \frac{T}{2}-t \right) \right]; i_{R2}\left( \frac{T}{2} \right)=i_{R1}\left( 0 \right)$ | (15) |
| --- | --- |

with (A10) will give us the input current (A17).

| $i_{1}\left( t \right)=\left\{ \begin{aligned} \frac{U_{L}}{2R_{Z}}-\frac{U_{L}}{2\omega L_{R0}}+\frac{U_{L}\pi}{\omega L_{R0}}\left( 1-cos\left( \omega t \right) \right)-\frac{U_{L}}{L_{R0}}t \\ \frac{U_{L}}{2R_{L}}-\frac{U_{m}\pi}{2\omega L_{R0}}+\frac{U_{L}\pi}{{\omega L}_{R0}}\left( 1+cos\left( \omega t \right) \right)+\frac{U_{L}}{L_{R0}}\left( \frac{T}{2}-t \right) \end{aligned}{; 0<t<\frac{T}{2} \atop; \frac{T}{2}<t<T} \right.$ | (A17) |
| --- | --- |

The fundamental harmonic can be further calculated using Fourier coefficients and is as (A18).

| $i_{1}\left( t \right)\approx\left[ \frac{2U_{L}}{\pi R_{L}}+\left( \frac{\pi-1}{\pi} \right)\frac{2U_{L}}{{\omega L}_{R0}R_{L}} \right]sin\left( \omega t \right)-\left( \frac{\pi^{2}-4}{\pi^{2}} \right)\frac{U_{L}\pi}{{\omega L}_{R0}}cos\left( \omega t \right)$ | (A18) |
| --- | --- |

Substituting into (A19), we find that there are two currents phase shifted by 90°, the first of which is purely ohmic, while the second is imaginary.

| $\frac{u_{1}}{i_{1}}\approx\frac{U_{1}}{I_{1}}\approx\frac{\frac{\pi U_{L}}{\sqrt{2}} sin(\omega t)}{\left[ \frac{2}{\sqrt{2}}\frac{U_{L}}{\pi R_{L}}+\left( \frac{\pi-1}{\pi} \right)\frac{2}{\sqrt{2}}\frac{U_{L}}{{\omega L}_{R0}R_{L}} \right]sin\left( \omega t \right)-\left( \frac{\pi^{2}-4}{\pi^{2}} \right)\frac{U_{L}\pi}{{\omega L}_{R0}\sqrt{2}}cos\left( \omega t \right)}$ | (A19) |
| --- | --- |

The rectifier behaves as a load composed of a parallel combination of the resistance and the inductance with respect to the supply voltage. This inductance will negatively affect the power factor of the rectifier and must be dealt with. From (A19) we determine the imaginary component of the impedance (A20).

| $\mathfrak{I}\left\{ \frac{U_{1}}{I_{1}} \right\}\approx\frac{\frac{\pi U_{L}}{\sqrt{2}}}{\left( \frac{\pi^{2}-4}{\pi^{2}} \right)\frac{U_{L}\pi}{{\omega L}_{R0}\sqrt{2}}}=\left( \frac{\pi^{2}}{\pi^{2}-4} \right){\omega L}_{R0}\Rightarrow L_{e}=\left( \frac{\pi^{2}}{\pi^{2}-4} \right)L_{R0}$ | (A20) |
| --- | --- |

Its effect can be fully compensated by an additional capacitor connected in parallel to the input terminals of the us-rectifier (A21).

| $C_{e}=\frac{1}{{2\omega}^{2}L_{e}}=\frac{1}{\omega^{2}\left( \frac{\pi^{2}}{\pi^{2}-4} \right)L_{R0}}$ | (A21) |
| --- | --- |

Similarly, we derive (A22).

| $L_{R0}=\frac{R_{Z}}{\omega}\left( \frac{\pi-1}{\pi} \right)$ | (A22) |
| --- | --- |
